# A commentary on “Comparative effectiveness of acupoint stimulation for preventing postoperative nausea and vomiting after general anesthesia: a network meta-analysis of randomized trials”

**DOI:** 10.1097/JS9.0000000000003047

**Published:** 2025-07-14

**Authors:** Liguo Li, Hongguang Fu, Chunxia Yu

**Affiliations:** a Institute of Rehabilitation Medicine, Henan Academy of Innovations in Medical Scienc, Zhengzhou, China; b Zhengzhou Health Vocational College, Zhengzhou, China; c Huanghe University of Science and Technology, Zhengzhou, China

*Dear Editor*,

We read with great interest the recent article titled “Comparative effectiveness of acupoint stimulation for preventing postoperative nausea and vomiting after general anesthesia: a network meta-analysis of randomized trials”^[^1^]^ published in the *International Journal of Surgery*. The purpose of this study is to thoroughly assess how well various acupoint stimulation methods work to prevent postoperative nausea and vomiting (PONV) following general anesthesia. The results showed that electroacupuncture and TEAS, with or without antiemetic, could both considerably lower the rates of postoperative nausea and vomiting following general anesthesia. However, a number of significant issues need to be addressed in future research.

First, the study covers a range of acupuncture methods, including acupressure, electro-acupuncture, and TEAS (Transcutaneous Electrical Acupuncture Stimulation). However, the operational parameters, including stimulation frequency and duration, are not standardized, which may lead to differences in therapeutic effects. For example, the intensity of TEAS stimulation or the selection of acupoints can vary across studies, making direct comparisons difficult. Furthermore, some studies combined antiemetic drugs, but the types and dosages of these drugs were not standardized, further increasing heterogeneity. While this diversity enriches the comparative dimensions, it may also obscure the true effectiveness of specific techniques. Future research should reduce heterogeneity through subgroup analysis or standardized protocols to enhance the comparability of results.

Second, the study lacks an assessment of long-term outcomes, such as 1 week following surgery, and instead concentrates on the incidence of postoperative PONV (Postoperative Nausea and Vomiting) between 24 and 72 hours. Additionally, the adverse reactions to acupuncture therapy, including skin irritation and pain, have not been systematically reported, and safety data are insufficient. Long-term efficacy and safety are crucial for the clinical application of non-drug therapies, especially for chronic conditions that require multiple interventions. Future studies should extend the follow-up period and meticulously document adverse events to comprehensively assess the benefit-risk ratio of the interventions. This limitation hinders the clinical guidance value of the research findings.

Third, the conclusions of network meta-analysis (NMA) rely on the consistency of indirect comparisons, even if NMA can compare different interventions. Statistical power may be impacted by some closed-loop studies’ limited sample sizes. Moreover, the impact of heterogeneity sources, such as surgical types and anesthesia methods, on the results has not been adequately explored. Although inconsistency tests were conducted, potential confounding factors, such as differences in patient baseline characteristics, may not have been fully controlled. Future research should further validate the robustness of the results through sensitivity analyses or regression models.

This study provides valuable evidence for the use of acupuncture in preventing postoperative nausea and vomiting (PONV), but it has limitations, including geographical limitations, gender bias, inadequate blinding, heterogeneity of interventions, lack of long-term data, and limitations in statistical methods. Future research should aim to increase sample diversity, standardize intervention measures, improve blinding design, and supplement with long-term follow-up and safety data to enhance the reliability and clinical applicability of the findings^[^2,3^]^. All of this was noted in relation to the TITAN guideline, which calls for transparency in the reporting of artificial intelligence^[^4^]^.

## Data Availability

No data was used in this Letter to the Editor.
